# Hetero-cellular interaction between cancer cells and stem cells in cancer metastasis

**DOI:** 10.1186/1742-4690-8-S1-A173

**Published:** 2011-06-06

**Authors:** Jamal El Saghir, Ali Bazarbachi, Youmna Kfoury, Marwan El-Sabban

**Affiliations:** 1 Department of Anatomy, Cell Biology, and Physiology, Department of Internal Medicine, Faculty of Medicine, American University of Beirut, Beirut, Lebanon

## Background

Metastasis, the most devastating aspect of cancer, is a multistep process by which cancer cells colonize secondary organ sites. Study of cellular and molecular determinants of cancer cells should be considered in the context of a complex micro-environment including hetero-cellular interactions that may influence disease progression. Stem cells, in the tumor micro-environment, are emerging as critical players in the acquisition of the invasive and metastatic phenotype.

## Aim

In this study, we assessed the role of paracrine and direct cell-cell interactions between human MSCs and Adult T-cell Leukemia/Lymphoma cells (HuT-102). We studied the transcriptomic modification induced by this reciprocal interaction and the role of connexin expression and gap junctional intercellular communication in modulating the potential of cancer cells to invade and metastasize.

## Methods

Trypan Blue exclusion assays cell counting and Real-Time PCR were performed to assess cancer cell proliferation and gene expression in indirect co-culture experiments. Endothelial monolayer invasion, cell communication assays and live imaging were performed to study the role of gap junctions in the direct cancer cell-stem cell interaction.

## Results

In a Trans-well co-culture system, MSCs (in insert) induced a decrease in HuT-102 proliferation while MSCs (in well) induced an increase in HuT-102 proliferation. VEGF and SDF-1 expression in MSCs (well) along with CXCR4 expression in HuT-102 (insert) was significantly up-regulated, leading to a marked increase in HuT-102 proliferation. Connexins were shown to be expressed in MSCs and HuT-102 and to facilitate the direct interaction between the two cell types. Induction of gap junctional intercellular communication is correlated with a 5-fold increase in HuT-102 trans-endothelial cell migration.

## Conclusion

Stem cells, in the tumor niche, and cancer cells reciprocally modulate each others phenotype and function, in a context-dependent manner, reinforcing the importance of tumor cellular micro-environment in cancer invasion and metastasis.

